# Supplementary material for: Impact of the COVID-19 Pandemic on Ambulatory Care Antibiotic Use in Hungary: A Population-Based Observational Study
Source: Antibiotics (Basel). 2023 May 27;12(6):970. doi: 10.3390/antibiotics12060970 (PMC10294918; doi:10.3390/antibiotics12060970)
Supplement: Supplementary file 1 [file antibiotics-12-00970-s001.zip › antibiotics-2401595-supplementary Tables S1.pdf]

	1. period	2. period	3.period	4. period	5. period
	**03.2020-05.2020**	**06.2020-10-2020**	**11.2020-04.2021**	**05.2021-10.2021**	**11.2021-03.2022**
**Kindergarden**	Close	Open	Open, but 03.08.-04.07. closed	Open	Open
**Elementary School**	Online education	Online education until end of the school year	under 14 year in-person education, over 14 year online education, but 03.08.-04.07. for everyone online education	First under 10 year in-person education, then for everyone	In-person education
**High School**	Online education	Online education until end of the school year	Online education	First just online, then in-person education	In-person education
**University**	Online education	Online education	Online education	Hibrid education	Hibrid education
**Hospitals**	Visiting ban	Resolve visiting	Visiting ban	Resolve visiting	Visiting ban
**Shops**	Open until 3 p.m., except: pharmacy, bakery, grocery store, market	Normal opening hours	Open until 7 p.m., than 03.08.-04.22. closed except: pharmacy, bakery, grocery store, market	Open first until 21:30, than normal opening hours	Normal opening hours
**Purchase period for elderly (over 65 year)**	9 a.m.-12 p.m.	-	9 a.m.- 11 a. m. (weekends 8 a.m.-10 a.m.)	-	-
**Restaurants, Confectioneries**	First open until 3 p.m., then just delivery	Opening first of the terraces, then inner places	Just delivery	Opening first of the terraces, then inner places with proof of vaccination card	Open for all
**Hotels**	Close	Receive guests	Closed, except: business, education	Receive guests only with proof of vaccination card	Receive guests only with proof of vaccination card
**Cinema, Museeum, Library, ZOO**	Close	Permited for all with 1.5 meter protecting distance	Close	Permited only with proof of vaccination card	Permited only with proof of vaccination card

**Sport facilities (Gym, Swimming pools, etc..)**	Close	Open with 1.5 meter protecting distance	Close	Permited only with proof of vaccination card	Permited only with proof of vaccination card
**Events – Inner spaces**	First maximum with 100 participants, than cancelled	Maximum with 200 participants	General ban on events	Parties permited only with proof of vaccination card Family events maximum 50 participants	Parties permited only with proof of vaccination card maximum with 200 participants
**Events - Outside**	First maximum with 500 participants, than cancelled	Maximum with 500 participants	General ban on events	Maximum with 500 participants	Maximum with 500 participants
**Cultural events**	Cancelled	Permited for all with 1.5 meter protecting distance	General ban on events	Permited only with proof of vaccination card	Permited only with proof of vaccination card
**Weddings, funerals**	Just with close family, weddings without party	Limited maximum 200 participants	General ban on events Just with close family, maximum 50 participatns, weddings without party	Maximum with 200 participants	Maximum with 200 participants
**Sport events**	First just without supporters/fans, than cancelled	Permited for all with 1.5 meter protecting distance		Permited only with proof of vaccination card	Permited only with proof of vaccination card
**Curfew**	Abode leaving just in case of emergency or work duties or to the shops	Suspended	First between 0 a.m. and 5 a.m, than between 8 p.m and 5 a.m.	First between 10 p.m. and 5 a.m., than suspended	Suspended
**Mask wearing (Inner spaces)**	Required in shops and on public transport	Required in shops, on public transport, in theatres and in customer offices	Required everywhere	-	Required in shops, in shopping centers, post offices, in theatres, in cinemas, in museeums, in sport events
**Mask wearing (Outside)**	-	-	Required first in outside events, than everywhere	-	-
