# Supplementary figures and images for: Impact of the COVID-19 Pandemic on Ambulatory Care Antibiotic Use in Hungary: A Population-Based Observational Study
Source: Antibiotics (Basel). 2023 May 27;12(6):970. doi: 10.3390/antibiotics12060970 (PMC10294918; doi:10.3390/antibiotics12060970)

## Beta-lactam antibacterials: Penicillins (J01C)

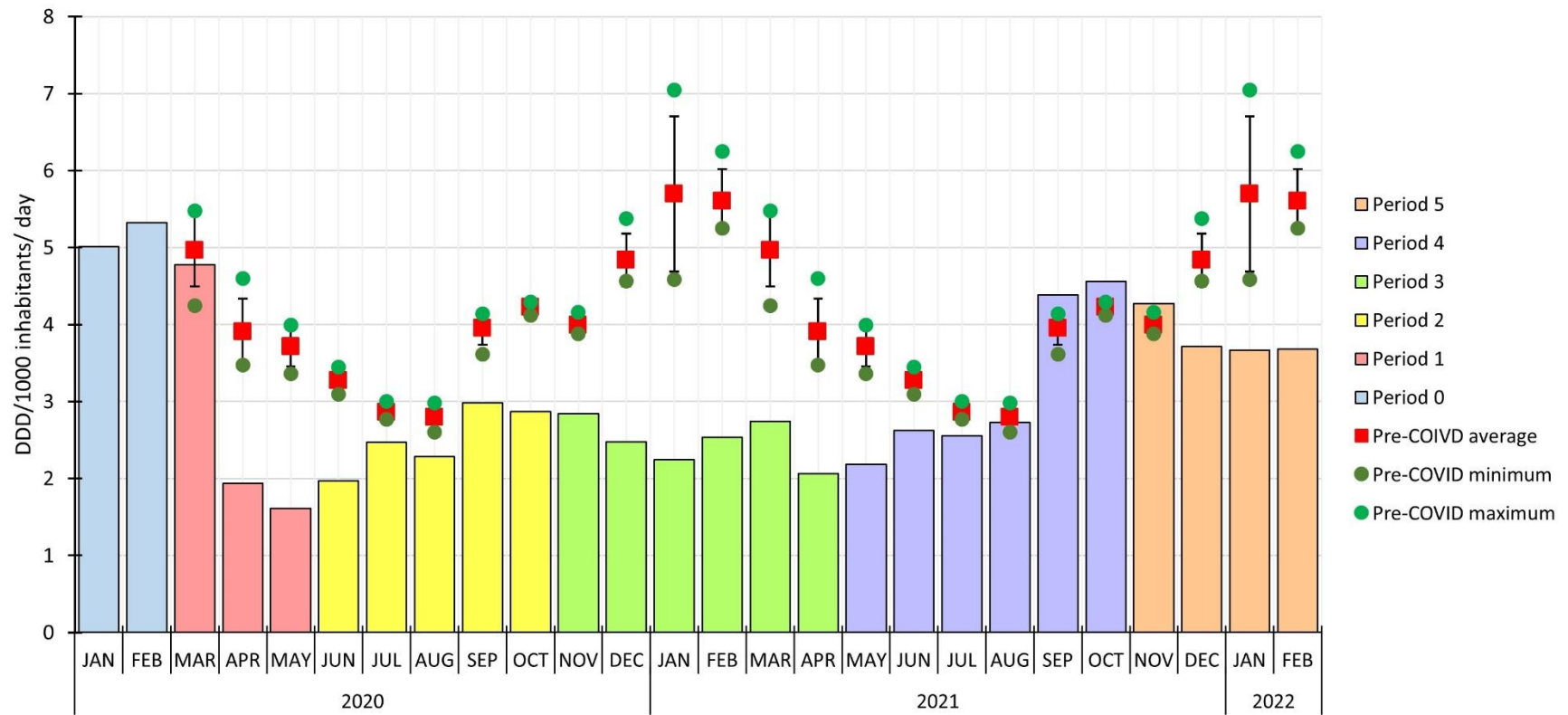

Supplement: Supplementary file 1 [file antibiotics-12-00970-s001.zip › antibiotics-2401595-supplementary Figure S1.pdf]

## Cephalosporins (J01D)

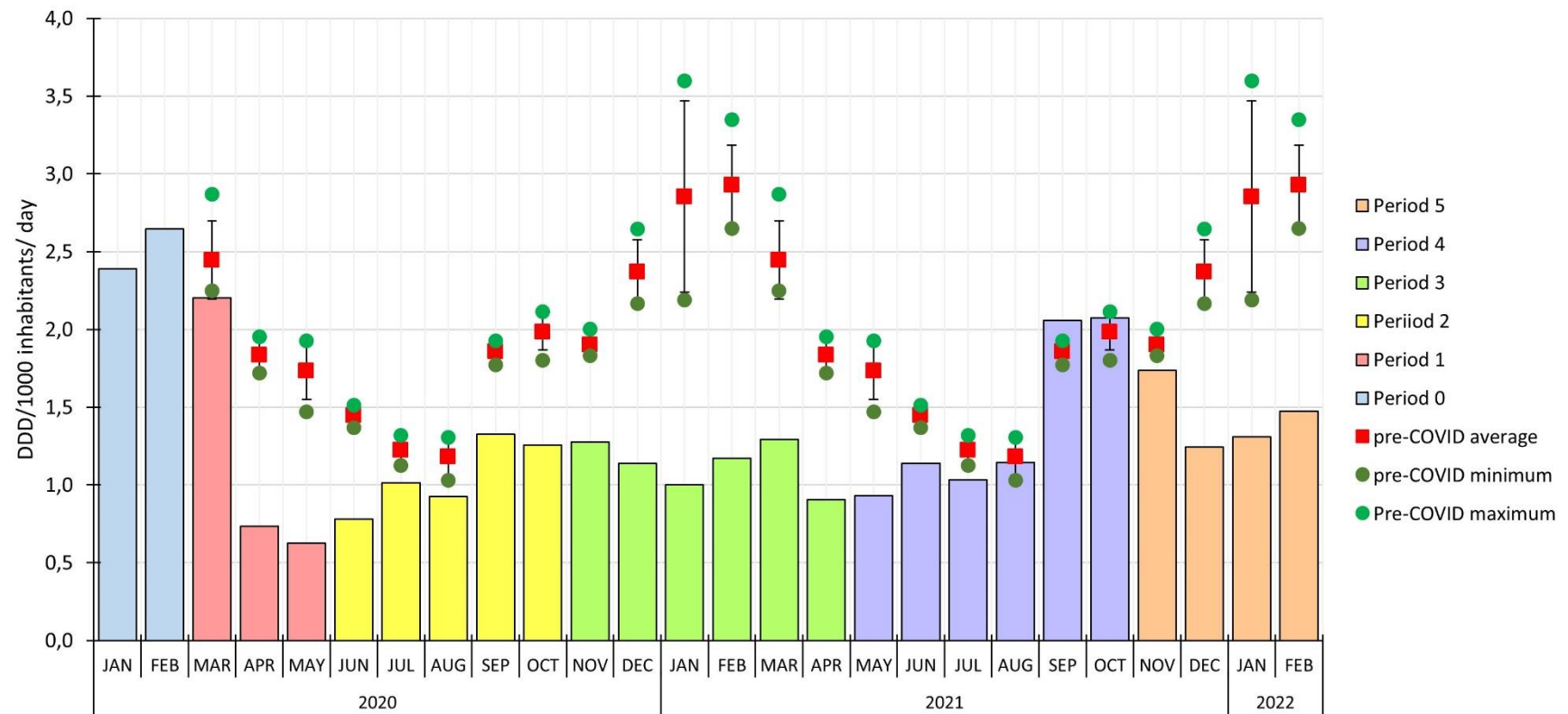

Supplement: Supplementary file 1 [file antibiotics-12-00970-s001.zip › antibiotics-2401595-supplementary Figure S2.pdf]

# Macrolides, liosaimdes (J01F)

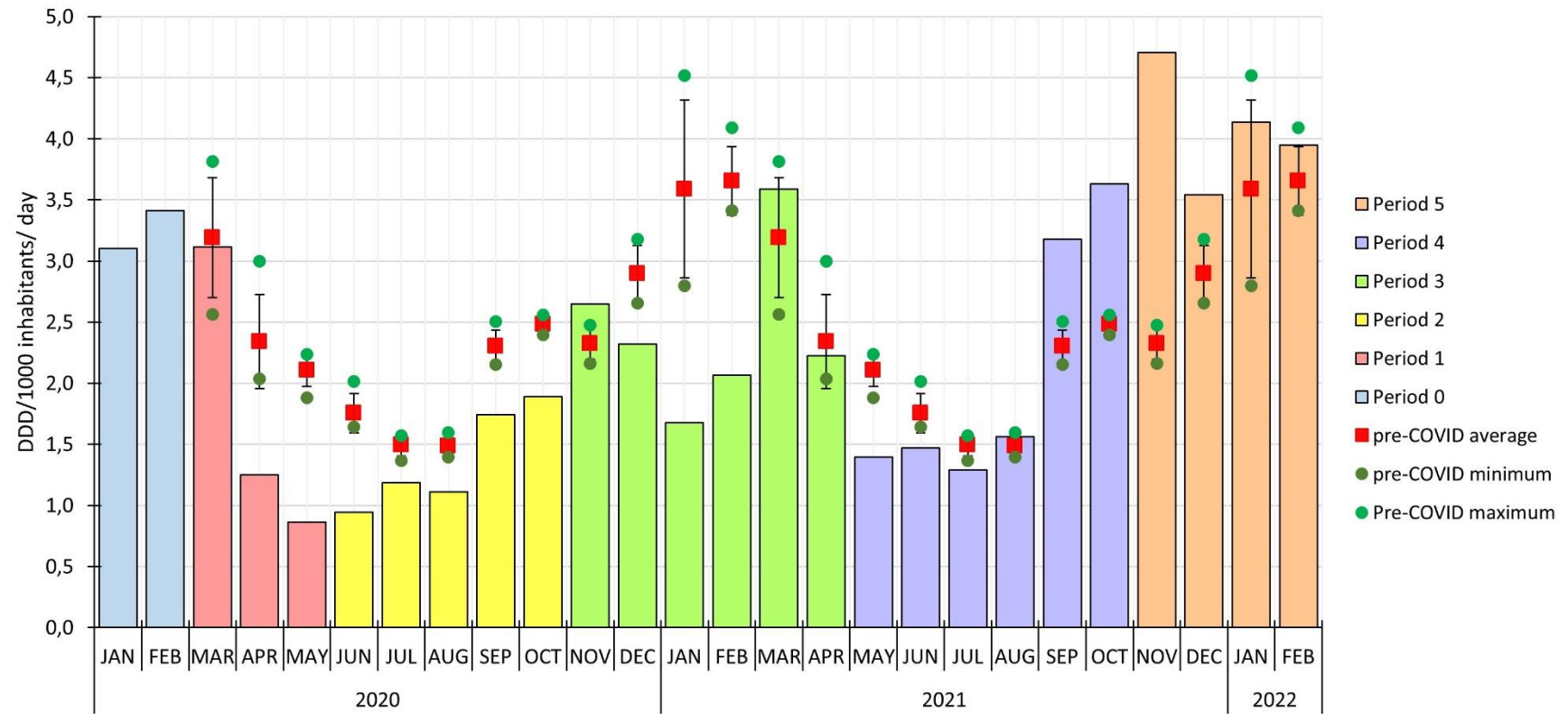

Supplement: Supplementary file 1 [file antibiotics-12-00970-s001.zip › antibiotics-2401595-supplementary Figure S3.pdf]

## Quinolone antibacterials (J01M)

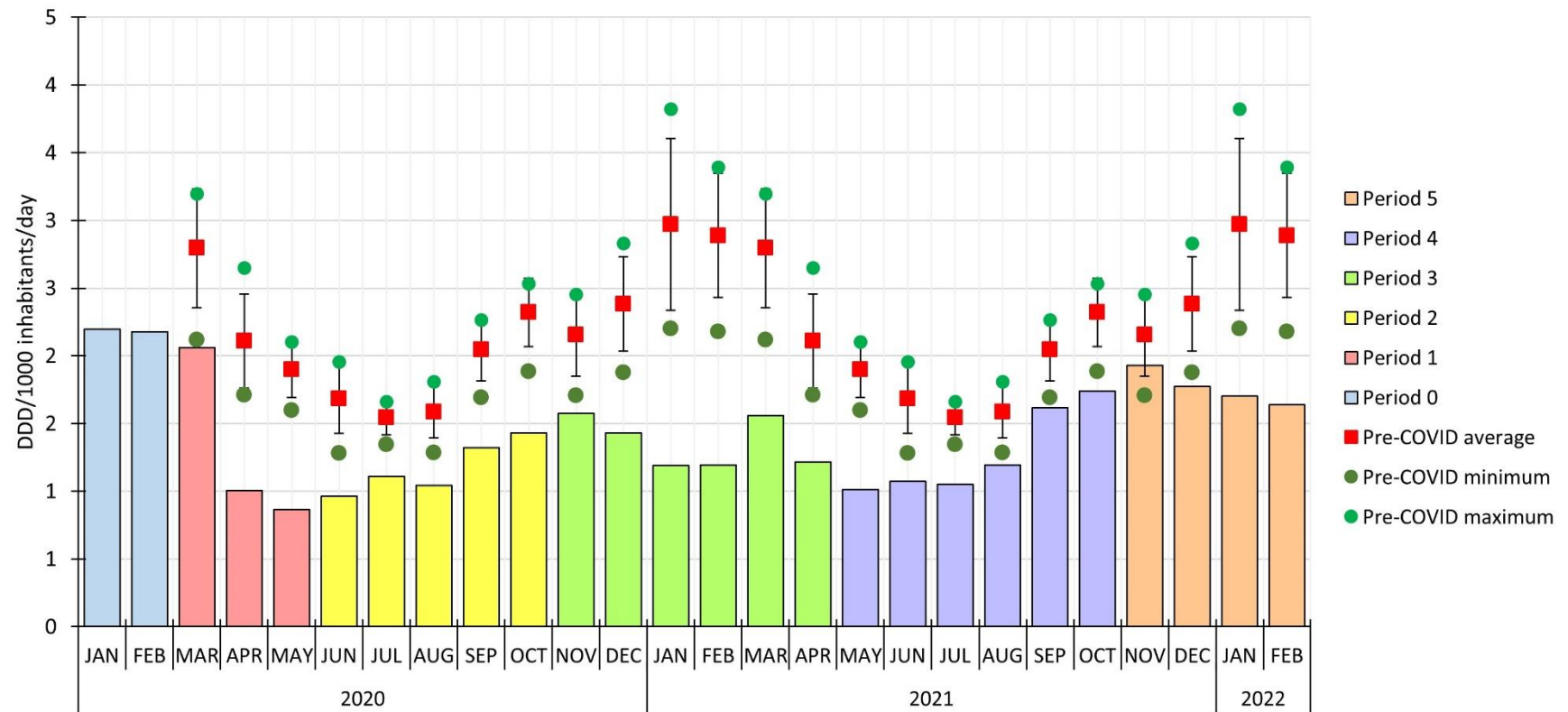

Supplement: Supplementary file 1 [file antibiotics-12-00970-s001.zip › antibiotics-2401595-supplementary Figure S4.pdf]
